# Qudit-Native Simulation of the Potts Model

**DOI:** 10.3390/e28020160

**Published:** 2026-01-31

**Authors:** Maksim A. Gavreev, Evgeniy O. Kiktenko, Aleksey K. Fedorov, Anastasiia S. Nikolaeva

**Affiliations:** Laboratory of Quantum Information Technologies, National University of Science and Technology “MISIS”, Moscow 119049, Russia

**Keywords:** Potts model, quantum simulation, qudits, trapped ions, Suzuki–Trotter decomposition

## Abstract

Simulating entangled, many-body quantum systems is notoriously hard, especially in the case of the high-dimensional nature of the underlying physical objects. In this work, we propose an approach for simulating the Potts model based on the Suzuki–Trotter decomposition that we construct for qudit systems. Specifically, we introduce two qudit-native decomposition schemes: (i) the first utilizes the Mølmer–Sørensen gate and additional local levels to encode the Potts interactions, while (ii) the second employs a light-shift gate that naturally fits qudit architectures. These decompositions enable a direct and efficient mapping of the Potts model dynamics into hardware-efficient qudit gate sequences for a trapped-ion platform. Furthermore, we demonstrate the use of a Suzuki–Trotter approximation with our evolution-into-gates framework for detecting the dynamical quantum phase transition. Our results establish a pathway toward qudit-based digital quantum simulation of many-body models and provide a new perspective on probing nonanalytic behavior in high-dimensional quantum many-body models.

## 1. Introduction

Quantum computational devices enable the exploration of entangled many-body quantum states that are believed to be difficult to analyze classically in a controllable manner [1]. The most well-developed digital model of quantum computing follows along the lines of classical information theory [2], so that the information units in the quantum domain are qubits that are quantum counterparts of classical information bits. However, underlying physical objects in physical systems that are used for quantum computing [3,4,5] are essentially multilevel [6], e.g., neutral atoms and trapped ions with a rich structure of energy levels (so that their use as qubits requires idealization). Since one of the key applications of quantum computing is the simulation of complex entangled many-body systems, the ability to manipulate multilevel states can be exploited to efficiently simulate quantum models consisting of high-dimensional objects, such as high-order spins [7].

We note that the use of multilevel systems, also known as qudits, in quantum computing is also of general interest in the context of a more efficient implementation of quantum algorithms (for a review, see Refs. [8,9]) Moreover, one can note significant progress in the realization of qudit-based processors with trapped ions [10,11], superconducting systems [12,13], and quantum light [14]. Several experiments have been performed using qudit processors for quantum simulations [15,16,17,18,19], for example, in the case of high-energy physics models [20].

A prominent example of a quantum many-body model with a wide range of applications is the Potts model [21,22,23], which is a generalization of the famous quantum Ising model (the Potts model is also related to the so-called chiral clock model [24,25,26]). The key feature of the Potts model is the ability to capture richer structures in quantum phase transitions [24,27,28], critical phenomena [28], exotic quasiparticle excitations (e.g., mesonic and baryonic) [29], properties of integrable lattice models [30], and entanglement structures [31]. However, as in the case of the Ising model beyond a certain number of spins, the Potts model becomes intractable with classical computational devices (we note that various approximate approaches such as Monte Carlo [32,33] and projected entangled-pair state (PEPS) [34] simulations have been demonstrated). We also note that transitions into complex $Z_{n}$-ordered phases, where excitations are evenly separated by n>2 sites, have been explored in experiments with programmable Rydberg quantum simulator [28]; the possibility to explore the critical properties of the three-state Potts model with fine-tuned pulses has been also mentioned.

In this work, we propose a way to study the Potts model using the Suzuki–Trotter (ST) decomposition that we construct for qudit systems. For this purpose, we introduce two new qudit-native decomposition schemes. The first scheme is based on the light-shift (LS) gate that is well suited to qudit architectures [35]. The second approach utilizes additional local levels to encode the Potts interactions. These decompositions allow one to map the Potts model dynamics into hardware-efficient gate sequences. As a target qudit hardware platform, we consider a trapped-ion platform, which is widely used for qudit-based simulations [15,16,36] and algorithm realizations [10,37,38]. We also conduct numerical experiments to demonstrate how gate decompositions can be used within the Suzuki–Trotter framework for dynamics simulation. We demonstrate how the presented decompositions can be used to observe a dynamical quantum phase transition in the Potts model.

The paper is organized as follows. In Section 2, we introduce the Potts model in its canonical formulation. Section 3 discusses the Suzuki–Trotter (ST) decomposition scheme. In Section 4, we present the qudit-native decompositions of the single- and two-qudit gates arising within the ST framework. Section 5 presents the numerical simulation results demonstrating a dynamical quantum phase transition (DQPT) in the Potts model. In Section 6, we compare qubit- and qudit-based quantum simulations from an information-theoretic perspective. We summarize our findings in Section 7.

## 2. Quantum Potts Model

The quantum Potts model generalizes the well-known transverse-field Ising model by extending the local Hilbert space from two to *q* internal states. Each site of the system is thus described by a *q*-dimensional qudit, and the model captures a wider range of symmetry-breaking and critical phenomena arising from the $Z_{q}$ discrete symmetry group. The Hamiltonian of the 1D quantum Potts chain can be written as

(1) $$\begin{array}{ccc} & & H=H^{I}+H^{L}, \end{array}$$

(2) $$\begin{array}{ccc} & & H^{I}=-J\sum_{\langle n,n^{\prime }\rangle }H_{n,n^{\prime }}^{I},\;H^{L}=-g\sum_{n}H_{n}^{L} \end{array}$$

(3) $$\begin{array}{ccc} & & H_{n,n^{\prime }}^{I}=\sum_{k=1}^{q-1}\Omega_{n}^{k}\Omega_{n^{\prime }}^{q-k}\;H_{n}^{L}=\sum_{k=1}^{q-1}\Gamma_{n}^{k}, \end{array}$$

where Ω and Γ are clock and shift operators respectively.

(4) $$\Omega =\left(\begin{array}{cccc} 1 & \; & & \\ \; & \omega & \; & \\ \; & \; & \ddots & \\ \; & \; & & \omega^{q-1} \end{array}\right),\;\Gamma =\left(\begin{array}{cc} \; & I_{q-1}\\ 1 & \end{array}\right),$$

where $\omega =e^{2\pi i/q}$ is the *q*th root of unity, and $I_{q-1}$ is the identity matrix of dimension q−1.

Clock and shift operators can be considered as high-dimensional generalizations of qubit Z,X operators. In the special case q=2, Ω and Γ reduce to the standard Pauli matrices $\sigma_{z}$ and $\sigma_{x}$, respectively, and the Hamiltonian becomes equivalent to the transverse-field Ising model.

The interplay between the interaction term, which energetically favors alignment of neighboring qudits, and the transverse mixing term, which induces transitions between local states, gives rise to a rich dynamical and equilibrium phase structure. For |g|≪|J|, the system exhibits a ferromagnetic phase characterized by ordered configurations with all qudits occupying the same internal state. In the opposite limit |g|≫|J|, the eigenstates become delocalized superpositions of all local levels, forming a disordered, quantum-paramagnetic phase.

From the perspective of quantum simulation, the Potts model represents an ideal benchmark for studying the effect of dimensionality in local Hilbert spaces and for exploring critical dynamics in multilevel quantum systems. The presence of non-commuting interaction and mixing terms makes the model analytically intractable in most regimes, especially out of equilibrium.

## 3. Suzuki–Trotter Decomposition

Digital quantum simulation provides a framework for approximating the continuous-time dynamics of quantum many-body systems by a discrete sequence of unitary gates that act on local subsystems [39]. The goal is to express the total time-evolution operator generated by a Hamiltonian *H* as a product of unitary operators that each act on a small subset of degrees of freedom.

Consider a general Hamiltonian Equation (1) written as a sum of interaction and local mixing terms where operators $H^{I},H^{L}$ typically do not commute with one another $[H^{I},H^{L}]\neq 0$. The first-order Lie–Trotter approximation replaces the global exponential with a product of local exponentials evaluated over a small time step τ=t/m:

(5) $$U\left(t\right)={\left[\exp\left(-i\tau H^{I}\right)\exp\left(-i\tau H^{L}\right)\right]}^{m}+O\left(\tau^{2}/m\right).$$

Higher-order Suzuki–Trotter formulas symmetrize exponentials to suppress this error to higher powers of τ at the expense of additional gate operations. This trade-off between accuracy and circuit depth plays a central role in practical implementations of digital quantum simulation. For the *q*-state Potts Hamiltonian, applying the second-order Suzuki–Trotter formula to this Hamiltonian gives

(6) $$U\left(t\right)={\left[U^{L}\left(\tau /2\right)U^{I}\left(\tau \right)U^{L}\left(\tau /2\right)\right]}^{m}+O\left(\tau^{3}/m^{2}\right),$$

where

(7) $$U^{L}\left(\tau \right)=\exp\left(-i\tau H^{L}\right),\;U^{I}\left(\tau \right)=\exp\left(-i\tau H^{I}\right).$$

Since each Hermitian operator under the exponential is a sum of commuting terms, we can expand each unitary into product

(8) $$\begin{array}{cc} \; & U^{I}\left(\tau \right)=\prod_{\langle n,n^{\prime }\rangle }U_{n,n^{\prime }}^{I}\left(\tau \right),\;U^{L}\left(\tau \right)=\prod_{n}U_{n}^{L}\left(\tau \right), \end{array}$$

(9) $$\begin{array}{cc} \; & U_{n,n^{\prime }}^{I}\left(\tau \right)=\exp\left(i\tau JH_{n,n^{\prime }}^{I}\right),\;U_{n}^{L}\left(\tau \right)=\exp\left(i\tau gH_{n}^{L}\right), \end{array}$$

where each exponential factor can be identified as an elementary evolution gate acting either on a pair of qudits or on a single qudit.

The total Trotterized evolution therefore consists of *m* sequential layers, each composed of parallel applications of $\exp(i\tau JH_{n,n^{\prime }}^{I})$ followed by $\exp(i\tau gH_{n}^{L})$. In qudit-based architectures such as trapped-ion or Rydberg-atom platforms, these gates correspond to experimentally realizable multilevel interactions and local drives. The Suzuki–Trotter decomposition thus bridges the gap between the continuous-time dynamics of the Potts Hamiltonian and a discrete gate-based realization, providing a mathematically controlled approximation whose fidelity can be systematically improved by increasing the approximation order or decreasing the step size τ.

## 4. Gate Decompositions

### 4.1. Single-Qudit Gate Decomposition

Within the Suzuki–Trotter framework, the non-diagonal part of the Potts Hamiltonian, $H^{L}$, generates local single-qudit transitions between internal qudit levels. The generalized shift operator Γ acts as Γ|m〉=|(m+1)modq〉 and satisfies the commutation relation ΓΩ=ωΩΓ with $\omega =e^{2\pi i/q}$.

The operator Γ is diagonal in the Fourier basis:

(10) $$\Gamma =F_{q}^{†}\;\Omega \;F_{q},$$

where $F_{q}$ is the discrete Fourier transform in a *q*-dimensional basis. Summing over *k* yields

(11) $$H_{n}^{L}=F_{q}^{†}\left(\sum_{k=1}^{q-1}\Omega^{k}\right)F_{q}=F_{q}^{†}DF_{q},$$

with D=diag(q−1,−1,…,−1). Substituting this diagonalization into the mixer evolution operator gives

(12) $$U_{n}^{L}\left(\tau \right)=F_{q}^{†}\exp\left(ig\tau \;D\right)F_{q}$$

We note that the diagonal phase gate can be implemented virtually [40]. Thus, the entire cost of realizing $U_{n}^{L}\left(\tau \right)$ is determined by the cost of implementing two discrete Fourier transforms.

A *q*-dimensional qudit Fourier transform $F_{q}$ can be decomposed into a sequence of native two-level rotations of the form $R^{ab}\left(\theta ,\phi \right)=\exp\left(-i\theta /2\left[\cos\left(\phi \right)\sigma_{x}^{ab}+\sin\left(\phi \right)\sigma_{y}^{ab}\right]\right)$ acting on pairs of basis levels, as shown in [41]. A standard construction requires at most $\frac{q(q-1)}{2}$ two-level rotations. Since the mixer gate requires two Fourier transforms, the total decomposition cost is q(q−1). Because the diagonal phase shift is virtual, this bound is tight, and the mixer gate requires exactly the same cost as two Fourier transforms.

### 4.2. Two-Qudit Gate Decompositions

#### 4.2.1. LS-Gate-Based Decomposition

We now focus on the decomposition of the two-qudit interaction term arising in the Potts Hamiltonian. The central object of interest is the interaction operator $H_{n,n^{\prime }}^{I}=\sum_{k=1}^{q-1}\Omega^{k}\Omega^{q-k}$. This operator describes the coupling between two qudits mediated through their “clock” operators, and it plays the role of an interaction term. To understand its structure, let us evaluate the matrix elements of $H_{n,n^{\prime }}^{I}$ in the product basis $\left\{|s,s^{\prime }\rangle \right\}$, for $s,s^{\prime }\in \left\{0,1,\ldots ,q-1\right\}$ for fixed $n,n^{\prime }$. We find

(13) $$\langle s,s^{\prime }|H_{n,n^{\prime }}^{I}|s,s^{\prime }\rangle =\sum_{k=1}^{q-1}\omega^{k(s-s^{\prime })}.$$

The summation above is a discrete Fourier sum over the phase difference between the two levels *s* and $s^{\prime }$. It evaluates to *q* when $s=s^{\prime }$ and to zero otherwise, since

(14) $$H_{n,n^{\prime }}^{I}|s,s^{\prime }\rangle =\left\{\begin{array}{cc} \left(q-1\right)|s,s^{\prime }, & s=s^{\prime }\\ -|s,s^{\prime }\rangle , & s\neq s^{\prime }. \end{array}\right.$$

Hence, the operator $H_{n,n^{\prime }}^{I}$ acts as a projector onto the subspace where both qudits occupy the same level. Explicitly, we can write

(15) $$H_{n,n^{\prime }}^{I}=q\sum_{s=0}^{q-1}\left|s,s\rangle \langle s,s\right|-I=q\Pi_{\mathrm{same}}-I,$$

where $\Pi_{\mathrm{same}\;}$ is the projector onto the symmetric subspace of equal-level states. The constant shift by −I contributes only a global phase in the time evolution and can therefore be neglected. The two-qudit time-evolution operator generated by $H_{n,n^{\prime }}^{I}$ during a time interval *t* reads

(16) $$U_{n,n^{\prime }}^{I}\left(\tau \right)=\exp\left(i\tau qJ\Pi_{\mathrm{same}}\right),$$

where *J* is the interaction strength. The exponential form shows that U(t) acts trivially on all states with $s\neq s^{\prime }$, while states with $s=s^{\prime }$ acquire a phase shift proportional to τqJ. The gate therefore applies a conditional phase depending on whether the two qudits are in the same state.

**Figure 1:**
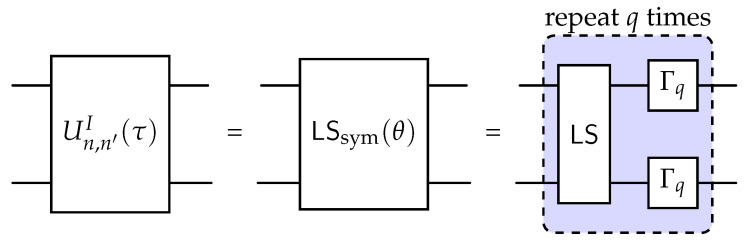
Two-qudit time-evolution operator implementation using light-shift (LS) gate.

Such a phase pattern corresponds exactly to the experimentally realized symmetric light-shift (LS) gate introduced for a trapped-ion qudit platform in [35] and obtained by symmetrizing its non-symmetric version LS (Figure 1). This gate acts as

(17) $${\mathrm{LS}}_{\mathrm{sym}}\left(\theta \right):\left\{\begin{array}{cc} |s,s\rangle \mapsto |s,s\rangle \\ |s,s^{\prime }\rangle \mapsto e^{i\theta }|s,s^{\prime }\rangle , & s\neq s^{\prime }, \end{array}\right.$$

and can equivalently be written as

(18) $${\mathrm{LS}}_{\mathrm{sym}}\left(\theta \right)=e^{i\theta }\exp\left(-i\theta \Pi_{\mathrm{same}}\right).$$

Comparing this with the expression for $U_{n,n^{\prime }}^{I}\left(t\right)$ above, we immediately identify the correspondence θ=−τqJ (for LS gate parameter tuning, see [35]’s supplementary materials). Hence, the LS gate provides a native realization of the two-qudit evolution operator required by the Potts model requiring $O(q^{2})$ single-qudit gates and O(q) two-qudit gates. Its diagonal structure ensures that the interaction is purely phase-based and does not mix different computational basis states, making it particularly suitable for Trotterized simulation schemes where diagonal and off-diagonal terms alternate.

#### 4.2.2. Decomposition Based on an Additional Level

In some architectures, direct implementation of the LS-type interaction may not be available. An alternative route is to exploit an extended Hilbert space containing an additional, auxiliary level. This additional level enables the realization of the projector $\Pi_{\mathrm{same}}$ using pairwise interactions between effective two-level systems.

Let each physical qudit possess one ancilla level, denoted |q〉, in addition to levels {|0〉,|1〉,…,|q−1〉}. The full local Hilbert space is therefore H=span{|0〉,|1〉,…,|q−1〉,|q〉}. Within each two-level subspace spanned by {|k〉,|q〉} we define Pauli operators

(19) $$\sigma_{z}^{k}=|k\rangle \langle k|-|q\rangle \langle q|,\;\sigma_{x}^{k}=|k\rangle \langle q|+|q\rangle \langle k|.$$

These operators satisfy the same algebra as standard Pauli matrices in their respective subspaces. Using these definitions, we introduce the operator

(20) $$\Pi_{n,n^{\prime }}=\sum_{k=0}^{q-1}\sigma_{z,n}^{k}\sigma_{z,n^{\prime }}^{k},$$

which acts jointly on two qudits *n* and $n^{\prime }$. Let us consider its action on the product basis states $|s,s^{\prime }\rangle$ within the logical subspace $s,s^{\prime }\in \left\{0,1,\ldots ,q-1\right\}$ for fixed $n,n^{\prime }$. For each pair $\left(s,s^{\prime }\right)$,

(21) $$\begin{array}{c} \sigma_{z,n}^{k}\sigma_{z,n^{\prime }}^{k}|s,s^{\prime }\rangle =\left(\delta_{s,k}-\delta_{s,q}\right)\left(\delta_{s^{\prime },k}-\delta_{s^{\prime },q}\right)|s,s^{\prime }\rangle =\delta_{s,k}\delta_{s^{\prime },k}|s,s^{\prime }\rangle , \end{array}$$

since $\delta_{s,q}=0$ and $\delta_{s^{\prime },q}=0$ in the logical subspace. Summing over *k* gives $\Pi_{n,n^{\prime }}|s,s^{\prime }\rangle =\delta_{s,s^{\prime }}|s,s^{\prime }\rangle$. Therefore, within the logical subspace, the operator $\Pi_{n,n^{\prime }}$ acts identically to the projector $\Pi_{\mathrm{same}}$. This observation allows us to replace $\Pi_{\mathrm{same}}$ by $\Pi_{n,n^{\prime }}$ in the desired evolution operator. The corresponding two-qudit unitary is then

(22) $$U_{n,n^{\prime }}^{I}\left(\tau \right)=\exp\left(i\theta \Pi_{n,n^{\prime }}\right)=\prod_{k=0}^{q-1}\exp\left(i\theta \sigma_{z,n}^{k}\sigma_{z,n^{\prime }}^{k}\right),$$

for θ=τqJ. The factorization in Equation (22) follows from the commutativity of the individual $\sigma_{z,n}^{k}\sigma_{z,n^{\prime }}^{k}$ terms acting on distinct subspaces. Each term represents a two-level conditional phase gate between the pair of subspaces ${\left\{|k\rangle ,|q\rangle \right\}}_{i}⊗{\left\{|k\rangle ,|q\rangle \right\}}_{j}$. To express these interactions in a more experimentally accessible form, it is convenient to rotate each subspace so that the coupling appears in the $\sigma_{x}$-basis. Defining

(23) $$V_{n}^{k}=\exp\left(-i\frac{\pi }{4}\sigma_{y}^{k}\right),$$

which maps $\sigma_{z}^{k}\mapsto \sigma_{x}^{k}$, we can rewrite the evolution as

(24) $$U_{n,n^{\prime }}^{I}\left(\tau \right)=\prod_{k=0}^{q-1}\left(V_{n}^{k}⊗V_{n^{\prime }}^{k}\right){\mathrm{MS}}_{n,n^{\prime }}^{k}\left(\theta \right)\left(V_{n}^{†,k}⊗V_{n^{\prime }}^{†,k}\right),$$

where

(25) $${\mathrm{MS}}_{n,n^{\prime }}^{k}\left(\theta \right)=\exp\left(i\theta \sigma_{x,n}^{k}\sigma_{x,n^{\prime }}^{k}\right)$$

denotes the Mølmer–Sørensen (MS) gate acting within the two-level subspace {|k〉,|q〉}; see Figure 2. The decomposition above shows that the entire two-qudit evolution $U_{n,n^{\prime }}^{I}\left(\tau \right)$ can be realized as a sequence of O(q) independent MS gates and O(q) single qudit gates, each acting on a distinct two-level manifold and conjugated by local rotations. In this way, the presence of a single auxiliary level per qudit suffices to emulate the multilevel interaction structure of the Potts Hamiltonian using only single-qudit rotations and widely used Mølmer–Sørensen two-qudit gates [10,11].

Conceptually, this construction demonstrates that even complex multiqudit projectors such as $\Pi_{\mathrm{same}}$ can be synthesized from simple pairwise couplings when an extended Hilbert space is available. It also highlights the modular nature of qudit control: by combining local subspace rotations and entangling gates, one can reproduce higher-dimensional interactions without requiring direct multilevel entanglement. This decomposition is therefore particularly suitable for scalable simulation architectures, as it enables implementation of the Potts-type two-qudit interactions entirely within the standard MS gate framework.

## 5. Qudit Simulation of the Potts Model: Dynamical Quantum Phase Transition

To assess the physical relevance of the presented decompositions, we performed a numerical simulation of the nonequilibrium dynamics governed by the qutrit (q=3) Potts Hamiltonian in a finite chain of N=6 qudits. The evolution was initialized in the ferromagnetic ground state of the interaction Hamiltonian, $|\psi_{0}\rangle ={⨂}_{i=1}^{N}{|0\rangle }_{i}$, and the subsequent unitary dynamics was driven by a sudden quench of the transverse parameter *g*, which activated the non-diagonal mixing term. The resulting evolution operator was applied iteratively for $N_{t}=t/\tau$ time steps, corresponding to total evolution time *t*. The system parameters were J=1/4,g=1.

A key observable characterizing the dynamical behavior is the return probability, or Loschmidt echo, defined as

(26) $$L\left(t\right)=|\langle \psi_{0}\left|\exp\left(-itH\right)\right|\psi_{0}{\rangle |}^{2},$$

which quantifies the overlap between the initial and time-evolved states. To make the analysis analogous to thermodynamic phase transitions, one introduces the rate function,

(27) $$\lambda \left(t\right)=-\frac{1}{N}\log L\left(t\right),$$

which plays the role of a dynamical free-energy density. Nonanalytic behavior or sharp peaks in λ(t) are interpreted as signatures of a dynamical quantum phase transition, reflecting critical changes in the temporal structure of the wave function under unitary evolution.

**Figure 3:**
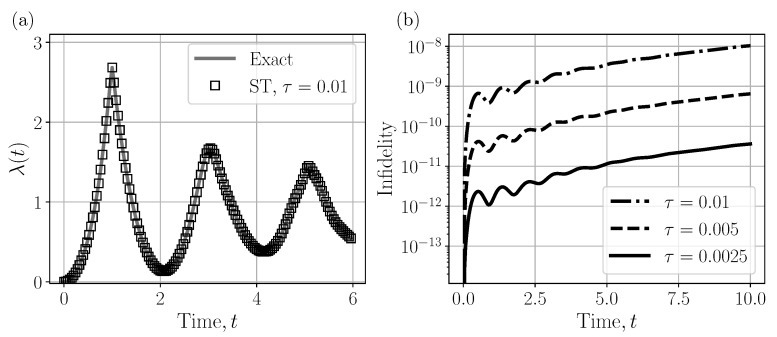
(**a**) Rate function for the simulated dynamics with the qutrit Potts Hamiltonian. (**b**) Infidelity caused by Trotterization errors.

Simulation results are summarized in Figure 3a, where the normalized logarithm of the return probability is plotted as a function of evolution time. In our simulations, we computed λ(t) using both the exact diagonalization of the full Hamiltonian and the Suzuki–Trotter decomposition with time step τ. The comparison demonstrated that even for moderate Trotter step sizes, the numerical approximation captured the essential dynamical features of the system. Figure 3b shows the infidelity plot between exact output state and approximate one. The characteristic cusps in the rate function appear at the same critical times as in the exact solution, indicating that the second-order Trotterized dynamics reproduced the DQPT signatures with high fidelity.

Physically, the appearance of nonanalyticities in λ(t) corresponds to critical points in the quantum state’s evolution, where the system undergoes coherent population transfer among macroscopically distinct configurations. In the q=3 Potts chain, this behavior reflects oscillatory competition between ordered domains and disordered superpositions induced by the transverse mixing. The observation of DQPTs in the simulated dynamics demonstrates that the qudit-based Suzuki–Trotter approach provides an efficient and accurate tool for studying nonequilibrium quantum phenomena beyond the qubit limit.

The simulation results remain valid for any two-qudit gate decomposition scheme, since both schemes are exact and do not introduce errors other than Trotterization. However, these two different schemes have different gate complexity. A comparison of the gate complexity of both schemes is given in Table 1. The gate counts are presented without transpilation. However, no straightforward gate simplifications are expected in the considered circuit’s construction.

These results validate the applicability of the Trotterized framework for future digital emulations of higher-dimensional Potts models and for benchmarking near-term qudit-based quantum processors. We also note that the simulation presented here with the specified parameters is available for existing trapped-ion quantum processors [10,11]. The numerical agreement with exact diagonalization establishes a quantitative foundation for scaling such simulations to larger systems where exact methods become intractable.

## 6. Qubits and Qudits

A fundamental question in digital quantum simulation is how the choice of local physical building blocks—qubits versus native qudits—affects the structure of quantum information generated during the simulation. Even when the target logical dynamics is identical, different physical representations may lead to qualitatively different patterns of correlations and entanglement, purely as a consequence of how local degrees of freedom are encoded.

In this section, we analyze these representation-dependent effects from an information-theoretic perspective. We focus on correlations that are not generated by the physical dynamics itself but are instead induced by the embedding of a higher-dimensional local Hilbert space into multiple lower-dimensional subsystems. To make this distinction explicit, we proceed in two steps. First, we study a concrete qubit encoding of a single qutrit and demonstrate that local logical operations inevitably generate intra-site correlations between the physical qubits. We then complement this microscopic picture with a general entropy-based argument, showing that such representation-induced entanglement is a generic feature of qubit encodings of qudits and scales with the local Hilbert-space dimension.

### 6.1. Representation-Induced Correlations in a Two-Qubit Encoding

To assess the intrinsic resource cost of encoding higher-dimensional local degrees of freedom into qubits, we compare a native qutrit realization of the q=3 quantum Potts model with a qubit-based encoding that reproduces the same logical dynamics. We employ the standard isometric embedding [42] of a single qutrit Hilbert space spanned by ${\left\{|\phi_{i}\rangle \right\}}_{i=0}^{2}$ into a two-qubit space $C^{3}↪{\left(C^{2}\right)}^{⊗2}$, defined as

(28) $$|\phi_{0}\rangle =|00\rangle ,\;|\phi_{1}\rangle =|01\rangle ,\;|\phi_{2}\rangle =|10\rangle ,$$

while the remaining computational basis state |11〉 spans an orthogonal leakage subspace. This mapping preserves the logical state of each *k*th qutrit,

(29) $$\rho_{k}\mapsto \rho_{A_{k}B_{k}}=\left(\begin{array}{cccc} \; & \; & & 0\\ \; & \rho_{k} & & 0\\ \; & \; & & 0\\ 0 & 0 & 0 & 0 \end{array}\right)$$

but replaces each local physical degree of freedom by a composite subsystem. This change in physical representation has a direct operational consequence: generic single-qutrit unitaries U∈SU(3) are mapped to entangling two-qubit operations. As a result, correlations between the two qubits encoding a single site are inevitably generated, even when the underlying Hamiltonian acts locally on a single site.

We interpret each logical site as a bipartite subsystem and quantify the resulting representation-induced correlations using the quantum mutual information,

(30) $$I\left(A_{k}:B_{k}\right)=S\left(\rho_{A_{k}}\right)+S\left(\rho_{B_{k}}\right)-S\left(\rho_{A_{k}B_{k}}\right),$$

where $\rho_{A_{k}}={\mathrm{Tr}}_{B_{k}}\left(\rho_{A_{k}B_{k}}\right)$ and $\rho_{B_{k}}={\mathrm{Tr}}_{A_{k}}\left(\rho_{A_{k}B_{k}}\right)$ are the reduced states of the *k*th encoded site, and $S\left(\varrho \right)=-\mathrm{Tr}\left[\varrho \log\varrho \right]$ denotes von Neumann’s entropy. By construction, $I(A_{k}:B_{k})\geq 0$ and vanishes if and only if $\rho_{A_{k}B_{k}}=\rho_{A_{k}}⊗\rho_{B_{k}}$, so it captures the total amount of classical and quantum correlations within the encoded site.

**Figure 4:**
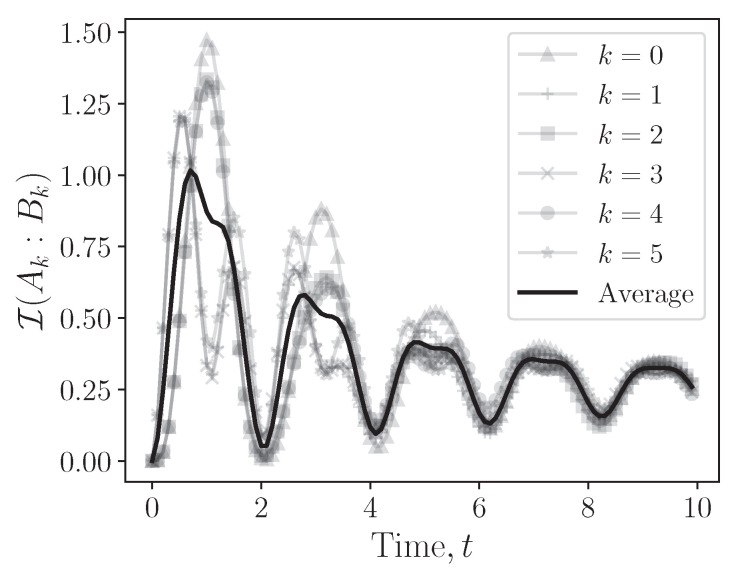
Mutual information $I(A_{i}:B_{i})$ between the two physical qubits encoding the *k*th qutrit under the mapping (28), for the same q=3, N=6 quench protocol as in Section 5. Thin curves show $I(A_{k}:B_{k})$ for individual sites k=0,…,5, while the thick curve corresponds to the site average $\bar{I}$.

As shown in Figure 4, the intra-site mutual information exhibits temporal oscillations, and its extrema closely track the oscillatory structure of the rate function λ(t). This synchronization indicates that the entanglement cost of the qubit encoding is dynamically activated by the same nonequilibrium processes that govern the global evolution. From a resource-theoretic perspective, $I(A_{k}:B_{k})$ quantifies the inevitable overhead that arises when simulating qudit dynamics with qubits: additional correlations need to be generated and maintained to reproduce the correct logical evolution. In a genuinely qudit-based architecture, this cost vanishes, as each site corresponds to a single physical system.

### 6.2. Representation-Induced Entropy Estimates

Understanding how representation change affects entanglement is essential for correctly interpreting entropy production, correlation growth, and resource costs in quantum simulations. In the following, we examine this issue in a single-qudit setting and show that qubit encodings generically induce entanglement that has no counterpart in the native qudit description.

Consider a single logical qudit with Hilbert space $H≅C^{q}$ prepared in a pure state |ψ〉. At the logical level, this system has no internal tensor-product structure and therefore carries no entanglement. We encode the qudit into $n=\lceil \log_{2}q\rceil$ physical qubits $C^{q}↪{\left(C^{2}\right)}^{⊗n}$, obtaining the encoded pure state. Fix a bipartition where *A* consists of a single physical qubit and *B* contains the remaining n−1 qubits, with dimensions $d_{A}=2$ and $d_{B}=2^{n-1}$. Consider the initial state |ψ〉 of a logical qudit as a state obtained by Haar-random unitary U∈SU(q) from the reference state. Then, following Page’s theorem [43] and using the asymptotics of the harmonic number, the mean entanglement entropy across the single-qubit cut is given by

(31) $$E_{U}\left[S\left(\rho_{A}\right)\right]=\ln2-2q^{-1}+O\left(q^{-2}\right),$$

i.e., the reduced state of a single physical qubit is asymptotically maximally mixed. Thus, even though the logical qudit is in a pure state, its qubit encoding necessarily exhibits nearly one bit of entanglement across every single-qubit bipartition.

This entropy is not generated by dynamics or interactions but arises purely from the qubit encoding of a high-dimensional logical degree of freedom, highlighting a fundamental distinction between native qudit descriptions and qubit-based representations.

## 7. Conclusions

In this work, we developed and analyzed a framework for simulating the dynamics of the *q*-state Potts model on qudit-based quantum architectures using the Suzuki–Trotter decomposition. The central idea is to map the continuous-time evolution under the many-body Hamiltonian onto a sequence of experimentally realizable qudit-native gates. We provided explicit decompositions for both single- and two-qudit unitaries, including a formulation based on the light-shift (LS) interaction and an alternative construction employing Mølmer–Sørensen gates and an auxiliary level to emulate projectors onto the logical subspace.

The numerical simulations for a three-level Potts chain demonstrated that the second-order Suzuki–Trotter approximation accurately reproduced the dynamical quantum phase transition observed in the exact unitary dynamics. The characteristic nonanalyticities in the rate function of the Loschmidt echo were clearly resolved, confirming that the qudit-based decomposition retained the essential nonequilibrium physics of the model.

These results establish a practical route for implementing multilevel quantum spin models on near-term qudit platforms, such as trapped ions or neutral atoms, where natural access to high-dimensional Hilbert spaces enables compact and hardware-efficient circuit realizations. The presented decompositions and their validation through exact numerical benchmarks form a foundation for extending digital simulation schemes to larger systems and higher-dimensional Potts models, bridging theoretical quantum many-body physics and experimental realizations of multilevel quantum dynamics.

## Figures and Tables

**Figure 2:**
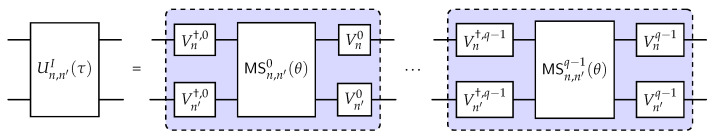
Two-qudit time-evolution operator implementation using ancilla levels.

**Table 1:** Gate complexity of single second-order Trotter step (N = 6) for different decomposition schemes.

	Section 4.2.1 (LS)	Section 4.2.2 (MS)
Two-qudit	15	15
Single-qudit	192	62
Depth	54	28

## Data Availability

Data are contained within the article.

## References

[1] Preskill J. (2012). Quantum computing and the entanglement frontier. arXiv.

[2] Brassard G., Chuang I., Lloyd S., Monroe C. (1998). Quantum computing. Proc. Natl. Acad. Sci. USA.

[3] Devoret M.H., Schoelkopf R.J. (2013). Superconducting circuits for quantum information: An outlook. Science.

[4] Temme K., Bravyi S., Gambetta J.M. (2017). Error Mitigation for short-depth quantum circuits. Phys. Rev. Lett..

[5] Neill C., Roushan P., Kechedzhi K., Boixo S., Isakov S.V., Smelyanskiy V., Megrant A., Chiaro B., Dunsworth A., Arya K. (2018). A blueprint for demonstrating quantum supremacy with superconducting qubits. Science.

[6] Kiktenko E.O., Pozhar N.O., Anufriev M.N., Trushechkin A.S., Yunusov R.R., Kurochkin Y.V., Lvovsky A.I., Fedorov A.K. (2018). Quantum-secured blockchain. Quantum Sci. Technol..

[7] Rico E., Dalmonte M., Zoller P., Banerjee D., Bögli M., Stebler P., Wiese U.J. (2018). SO(3) “nuclear physics” with ultracold gases. Ann. Phys..

[8] Kiktenko E.O., Nikolaeva A.S., Fedorov A.K. (2025). Colloquium: Qudits for decomposing multiqubit gates and realizing quantum algorithms. Rev. Mod. Phys..

[9] Nikolaeva A.S., Kiktenko E.O., Fedorov A.K. (2024). Efficient realization of quantum algorithms with qudits. EPJ Quantum Technol..

[10] Zalivako I.V., Nikolaeva A.S., Borisenko A.S., Korolkov A.E., Sidorov P.L., Galstyan K.P., Semenin N.V., Smirnov V.N., Aksenov M.A., Makushin K.M. (2025). Towards multiqudit quantum processor based on a ^171^Yb^+^ ion string: Realizing basic quantum algorithms. Quantum Rep..

[11] Ringbauer M., Meth M., Postler L., Stricker R., Blatt R., Schindler P., Monz T. (2022). A universal qudit quantum processor with trapped ions. Nat. Phys..

[12] Hill A.D., Hodson M.J., Didier N., Reagor M.J. (2021). Realization of arbitrary doubly-controlled quantum phase gates. arXiv.

[13] Nguyen L.B., Goss N., Siva K., Kim Y., Younis E., Qing B., Hashim A., Santiago D.I., Siddiqi I. (2024). Empowering a qudit-based quantum processor by traversing the dual bosonic ladder. Nat. Commun..

[14] Chi Y., Huang J., Zhang Z., Mao J., Zhou Z., Chen X., Zhai C., Bao J., Dai T., Yuan H. (2022). A programmable qudit-based quantum processor. Nat. Commun..

[15] Kazmina A.S., Zalivako I.V., Borisenko A.S., Nemkov N.A., Nikolaeva A.S., Simakov I.A., Kuznetsova A.V., Egorova E.Y., Galstyan K.P., Semenin N.V. (2024). Demonstration of a parity-time-symmetry-breaking phase transition using superconducting and trapped-ion qutrits. Phys. Rev. A.

[16] Meth M., Zhang J., Haase J.F., Edmunds C., Postler L., Jena A.J., Steiner A., Dellantonio L., Blatt R., Zoller P. (2025). Simulating two-dimensional lattice gauge theories on a qudit quantum computer. Nat. Phys..

[17] Chizzini M., Tacchino F., Chiesa A., Tavernelli I., Carretta S., Santini P. (2024). Qudit-based quantum simulation of fermionic systems. Phys. Rev. A.

[18] Chicco S., Allodi G., Chiesa A., Garlatti E., Buch C.D., Santini P., De Renzi R., Piligkos S., Carretta S. (2024). Proof-of-Concept Quantum Simulator Based on Molecular Spin Qudits. J. Am. Chem. Soc..

[19] Jiang J., Klco N., Di Matteo O. (2025). Non-Abelian dynamics on a cube: Improving quantum compilation through qudit-based simulations. Phys. Rev. D.

[20] Bauer C.W., Davoudi Z., Balantekin A.B., Bhattacharya T., Carena M., de Jong W.A., Draper P., El-Khadra A., Gemelke N., Hanada M. (2023). Quantum simulation for high-energy physics. PRX Quantum.

[21] Cheng B., Lin M., Huang G., Li Y., Ji B., Genin G.M., Deshpande V.S., Lu T.J., Xu F. (2017). Cellular mechanosensing of the biophysical microenvironment: A review of mathematical models of biophysical regulation of cell responses. Phys. Life Rev..

[22] Szabó G., Borsos I. (2016). Evolutionary potential games on lattices. Phys. Rep..

[23] Li M., Liu R.R., Lü L., Hu M.B., Xu S., Zhang Y.C. (2021). Percolation on complex networks: Theory and application. Phys. Rep..

[24] Samajdar R., Choi S., Pichler H., Lukin M.D., Sachdev S. (2018). Numerical study of the chiral $Z_{3}$ quantum phase transition in one spatial dimension. Phys. Rev. A.

[25] Yoo Y., Swingle B. (2024). Temperature dependence of energy transport in the $Z_{3}$ chiral clock model. Phys. Rev. B.

[26] Mahyaeh I., Ardonne E. (2018). Exact results for a $Z_{3}$-clock-type model and some close relatives. Phys. Rev. B.

[27] Bernien H., Schwartz S., Keesling A., Levine H., Omran A., Pichler H., Choi S., Zibrov A.S., Endres M., Greiner M. (2017). Probing many-body dynamics on a 51-atom quantum simulator. Nature.

[28] Keesling A., Omran A., Levine H., Bernien H., Pichler H., Choi S., Samajdar R., Schwartz S., Silvi P., Sachdev S. (2019). Quantum Kibble–Zurek mechanism and critical dynamics on a programmable Rydberg simulator. Nature.

[29] Liu F., Whitsitt S., Bienias P., Lundgren R., Gorshkov A.V. (2020). Realizing and Probing baryonic excitations in rydberg atom arrays. arXiv.

[30] Martin P.P. (1991). Potts Models and Related Problems in Statistical Mechanics.

[31] Lotkov A.I., Gritsev V., Fedorov A.K., Kurlov D.V. (2022). Floquet integrability and long-range entanglement generation in the one-dimensional quantum Potts model. Phys. Rev. B.

[32] Pan C. (1992). Monte Carlo simulation for the quantum q-state Potts model. J. Magn. Magn. Mater..

[33] Wen Z.B., Hou Z.L., Fu X.J. (2011). Monte carlo simulation of the potts model on a dodecagonal quasiperiodic structure. Chin. Phys. Lett..

[34] Śmierzchalski T., Dziubyna A.M., Jałowiecki K., Mzaouali Z., Pawela Ł., Gardas B., Rams M.M. (2025). SpinGlassPEPS.jl: Tensor-network package for Ising-like optimization on quasi-two-dimensional graphs. SoftwareX.

[35] Hrmo P., Wilhelm B., Gerster L., van Mourik M.W., Huber M., Blatt R., Schindler P., Monz T., Ringbauer M. (2023). Native qudit entanglement in a trapped ion quantum processor. Nat. Commun..

[36] Joshi R., Louw J.C., Meth M., Osborne J.J., Mato K., Su G.X., Ringbauer M., Halimeh J.C. (2025). Probing hadron scattering in lattice gauge theories on qudit quantum computers. arXiv.

[37] Low P.J., Zutt N.C.F., Tathed G.A., Senko C. (2025). Quantum logic operations and algorithms in a single 25-level atomic qudit. arXiv.

[38] Nikolaeva A.S., Zalivako I.V., Borisenko A.S., Semenin N.V., Galstyan K.P., Korolkov A.E., Kiktenko E.O., Khabarova K.Y., Semerikov I.A., Fedorov A.K. (2025). Scalable improvement of the generalized toffoli gate realization using trapped-ion-based qutrits. Phys. Rev. Lett..

[39] Lloyd S. (1996). Universal Quantum Simulators. Science.

[40] McKay D.C., Wood C.J., Sheldon S., Chow J.M., Gambetta J.M. (2017). Efficient Z gates for quantum computing. Phys. Rev. A.

[41] Drozhzhin D.A., Kiktenko E.O., Fedorov A.K., Nikolaeva A.S. (2026). Transition-aware decomposition of single-qudit gates. Entropy.

[42] López-Saldívar J.A., Castaños O., Nahmad-Achar E., López-Peña R., Man’ko M.A., Man’ko V.I. (2018). Geometry and entanglement of two-qubit states in the quantum probabilistic representation. Entropy.

[43] Page D. (1993). Average entropy of a subsystem. Phys. Rev. Lett..

